# Surgical Duration Implicated in Major Postoperative Complications in Total Hip and Total Knee Arthroplasty: A Retrospective Cohort Study

**DOI:** 10.5435/JAAOSGlobal-D-20-00043

**Published:** 2020-11-04

**Authors:** Mark D. Orland, Remy Y. Lee, Edmund E. Naami, Michael J. Patetta, Awais K. Hussain, Mark H. Gonzalez

**Affiliations:** From the Department of Orthopaedic Surgery, University of Illinois, Chicago, IL.

## Abstract

Total hip and knee arthroplasties are two of the most commonly performed orthopaedic surgeries and are expected to increase in incidence in the coming decades. We sought to examine whether the duration of these procedures is related to various postoperative complications using data from 2010 to 2017 from the American College of Surgeons National Surgical Quality Improvement Program database.

**Methods:**

The American College of Surgeons National Surgical Quality Improvement Program database was queried for patients undergoing total hip and knee arthroplasty by their respective Current Procedural Terminology codes. Operation time was stratified into four quartiles with equal sample sizes in each quartile for total hip and knee arthroplasty separately. The first quartile of surgical times was used as the control to which the other three quartiles were compared. Multivariate logistic regression analysis was performed on all samples that accounted for possible covariates, totaling 119,076 patients for total hip and 189,297 for total knee arthroplasty.

**Results:**

The third and fourth quartiles of total hip and total knee arthroplasty were markedly associated with higher incidences of wound complications, particularly infection and dehiscence. In addition, prolonged total hip arthroplasty was associated with a markedly higher rate of urinary tract infections for the third and fourth quartiles, and deep vein thrombosis in the fourth quartile.

**Conclusions:**

The surgical duration of total hip and knee arthroplasties is an independent risk factor for wound complications and several other important postoperative complications. Therefore, extensive preoperative planning and postoperative prophylactic measures should be performed to minimize patient morbidity and reduce hospital costs.

Surgical duration for joint arthroplasty is affected by a variety of factors including the surgeon's skill and experience, case complexity, and operating room efficiency.^1,2^ Currently, more than one million total hip and knee arthroplasties are performed each year in the United States with approximately seven million Americans living with a hip or knee arthroplasty.^3,4^ Predictive models have shown that by 2040, approximately five million total hip and knee arthroplasties will be performed each year with increased prevalence in both sexes and in all age groups.^5^ Given the projected increase in the frequency of these common procedures, thorough analysis of these operations and their complications is necessary to minimize both risk to patients and healthcare costs. There is consensus in the literature that both mild and major postoperative complications markedly increase hospital costs. However, there is insufficient literature investigating surgical time as a risk factor for these complications.^6^

Although certain studies have shown that surgical time has no impact on the durability of the prosthesis used in total knee and hip arthroplasties, others have implicated prolonged surgical duration as a cause of postoperative complications.^7-12^ This leads to extended hospitalizations and additional surgeries.^12^ The goal of this study was to determine whether operating times are a risk factor for postoperative complications in patients undergoing total hip (THA) or total knee arthroplasty (TKA).

## Methods

### Data Collection

This was a retrospective analysis of prospectively collected data from 2010 to 2017 in the American College of Surgeons National Quality Improvement Program (ACS-NSQIP) database. The national database is a collection of more than 300 variables containing patient demographics, preoperative comorbidities, perioperative complications, and postoperative complications.^13^ The data were collected from over 500 hospitals across the United States.^14^

### Data Selection

Based on the data in the ACS-NSQIP, only patients undergoing primary total hip or total knee arthroplasty procedures were included. These patients were determined based on these CPT codes, 27130 for THA and 27447 for TKA.^15,16^ To identify confounding variables in subsequent analyses, data were filtered to include samples that accounted for the following variables: sex, race, age, cardiovascular disease, smoking status, abnormal kidney function (as indicated by dialysis use), thrombocytopenia (platelet count <150,000 cells/mm^3^), anemia (hematocrit <36% for women and <39% for men, World Health Organization), obesity (body mass index >30 kg/m^2^), diabetes, pulmonary comorbidities, bleeding disorders, steroid or immunosuppressant use, dyspnea at rest, and preoperative blood transfusion. Only samples with a complete record of the aforementioned variables were included in the analyses.

### Data Stratification

The data for THA and TKA were separately analyzed. The samples were sorted by surgical duration, defined by ACS-NSQIP as the time from initial incision to skin closure, and divided into four quartiles. The stratification can be seen in Table 1 below. Age was also stratified into four quartiles using the same methodology.

**Table 1:** Surgical Duration Quartiles for Total Hip and Knee Arthroplasty

Arthroplasty Procedure	Quartile	Time Range (min)
Total hip	First	0-68
	Second	69-86
	Third	87-110
	Fourth	≥111
Total knee	First	0-70
	Second	71-86
	Third	87-107
	Fourth	≥108

### Thirty-Day Postoperative Complications

Postoperative complications that occurred within 30 days of the surgery were found in the ACS-NSQIP database. These included wound complications (wound infection, surgical site infection, organ space infection, and wound dehiscence), clotting complications (deep vein thrombosis, venous thromboembolism, myocardial infarction, and stroke), blood transfusions, unplanned intubation, urinary tract infections, and major systemic complications including septic shock and cardiac arrest.

### Statistical Analysis

All analyses were done with the R software, version 3.6.1.^17^ Statistical significance was set as *P* < 0.05. A multiple logistic regression model was used to test the effect of surgical duration on postoperative complications, with patients in the first quartile set as the control group against the other three quartile groups. This analysis method was selected because the surgical duration was stratified as a categorical variable, whereas a linear regression model would better accommodate a continuous variable.^18^ Patient demographics and comorbidities that were markedly associated with surgical duration were included as covariates in the model. Odds ratios (ORs) and 95% confidence intervals were reported to measure the effect of surgical duration on postoperative complications.

## Results

### Total Hip Arthroplasty

Pearson χ^2^ univariate analysis was used to determine patient preoperative covariates to ensure every patient analyzed had all covariates accounted for 119,076 patients who underwent THA were thus analyzed using multiple logistic regression. The following were significant covariates that analyzed patients included: sex, race, smoking status, anemia, age, obesity, diabetes, pulmonary comorbidities, dyspnea, and preoperative transfusion. Full results of the univariate analysis can be found in Supplemental Table 1, http://links.lww.com/JG9/A91.

The first quartile of operative times for THA were from 0 to 68 minutes; the second quartile included operative times from 87 to 110 minutes; the third quartile included operative times from 87 to 110 minutes; the fourth quartile included operative times longer than 111 minutes.

The second quartile of surgical times was associated with significantly more postoperative blood transfusions (*P* < 0.001, OR, 1.23, 95% confidence interval, 1.15-1.33). There were no other significant postoperative complications associated with this quartile.

The third quartile of surgical times was associated with a significantly increased incidence of wound complications including surgical site infection, deep incisional infection, wound infection, organ space infection, and sepsis (all with *P* < 0.05, OR >1). In addition, a markedly higher rate of urinary tract infections and postoperative transfusions occurred in patients with third quartile procedure duration.

The fourth quartile of total hip procedural times was also markedly associated with the same complications as the third quartile. In addition, patients presented with markedly increased rates of deep vein thrombosis and wound dehiscence complications (all *P* < 0.05, OR >1). Full results for all quartiles can be found in Supplemental Table 2, http://links.lww.com/JG9/A92.

### Total Knee Arthroplasty

Pearson χ^2^ univariate analysis was used to determine patient preoperative covariates to ensure every patient analyzed had all covariates accounted for 189,297 patients who underwent TKA were thus analyzed. The following were notable covariates that analyzed patients included sex, race, smoking, anemia, age, obesity, diabetes, pulmonary comorbidity, dyspnea, and preoperative transfusion. The same covariates were notable in TKA. Full results of the univariate analysis can be found in Supplemental Table 3, http://links.lww.com/JG9/A93.

The first quartile of surgical times for TKA were from 0 to 70 minutes; the second quartile included surgical times from 71 to 86 minutes; the third quartile included surgical times from 87 to 107 minutes; the fourth quartile included surgical times longer than 108 minutes.

The second quartile of surgical times for TKA was insignificant for postoperative complication incidence when compared with the first quartile.

The third quartile of surgical times was found to have markedly increased wound complications as compared with the first quartile. More surgical site infections, deep site infections, organ space infections, wound dehiscence, and wound infections (*P* < 0.05, OR >1) were observed. For surgical times longer than 111 minutes, all complications listed in third quartile remained notable, and an increased incidence of deep vein thrombosis (*P* < 0.05) was noted. Full results can be found in Supplemental Table 4, http://links.lww.com/JG9/A94.

## Discussion

Although patients undergoing THA or TKA typically understand the serious complications associated with these procedures including death and serious impairment, specifics including the risk of wound complication are not often discussed during preoperative evaluations.^19,20^ Our results showed that patients who underwent THA or TKA with surgical durations longer than 87 minutes were at a markedly higher risk for wound complications. In addition, the risk of sepsis was markedly increased in THA for longer procedure times. Similar studies examining surgical times and the impact on total joints complications have been conducted by Duchmann et al and Bohl et al. Although both demonstrate outliers in surgical times lead to poor results, Duchmann set complication cutoffs anecdotally at less than 1 hour and greater than 2 hours in his analysis. By analyzing by quartiles of surgical time, our study was able to better quantify relationships for cutoff risk, demonstrating additional risks between 87 and 120 minutes. Bohl et al captured these complications and quantified an increased risk in correspondence to 15-minute intervals. However, their analysis examined THAs and TKAs together, rather than independently as done in our analysis.

Given these findings, improving preoperative planning, operating room team cohesion, and protocols to treat wound complications may help mitigate patient morbidity. Studies have shown that increased preoperative planning for elective TKAs led to shorter surgical time when compared with nonelective TKAs. This resulted in reduced postoperative complication rates.^21^ In addition, new technological advances including 3D reconstructions from radiographs enhance preoperative planning and patient outcomes.^22,23^ In addition, multiple studies have demonstrated implementation of multidisciplinary teams lead to better patient outcomes, which were even further enhanced through team simulations.^24-26^ Multidisciplinary teams have also been shown to improve cohesion and minimize interruptions to the operating room.^24,27^ Given the average operating room is interrupted 9.92 times per hour, this can greatly reduce surgical time.^27^

Finally, increased surgical experience can improve surgeons' technical abilities and thus operating room efficiency.^28^ In addition to conventional practice methods, virtual reality (VR) simulations have increased surgical residents' mastery of surgical techniques.^29^ VR can also be used to familiarize the operating room staff with the steps and instruments used during operations to best assist the surgeon.^30^ Comprehensive perioperative antibiotic regimen may also help mitigate risk of infection in cases with long operating times.^31^ In addition, strict adherence to the World Health Organization^32^ global guidelines to prevent infection can help lower the risk of surgical site infection.

The main limitations of this study stemmed from multicentered, large numbered origin. First, we cannot measure how volume and surgical experience may confound the interpretations. It is reasonable to think a more experienced surgeon would have less complications than a less experienced surgeon, even if surgical times were equivalent. Similarly, there is inherent variation in the complexity of each TKA and THA case that affects the duration of surgery.

## Conclusions

Longer surgical durations in THA and TKA are associated with a markedly higher risk of wound complications. Enhancing operating room efficiency through extensive preoperative planning, communication, and practice can reduce the surgical duration. In cases with longer anticipated surgical times, appropriate perioperative measures should be taken to minimize patient morbidity and reduce hospital costs.

## Supplementary Material

SUPPLEMENTARY MATERIAL
