# Supplementary material for: Surgical Duration Implicated in Major Postoperative Complications in Total Hip and Total Knee Arthroplasty: A Retrospective Cohort Study
Source: J Am Acad Orthop Surg Glob Res Rev. 2020 Nov 4;4(11):e20.00043. doi: 10.5435/JAAOSGlobal-D-20-00043 (PMC7643914; doi:10.5435/JAAOSGlobal-D-20-00043)
Supplement: SUPPLEMENTARY MATERIAL [file jagrr-4-e20.00043-s001.docx]

| **Preoperative Variable** | **Quartile 1** | | **Quartile 2** | | **Quartile 3** | | **Quartile 4** | | **P-value** |
| --- | --- | --- | --- | --- | --- | --- | --- | --- | --- |
|  | Number | Percent | Number | Percent | Number | Percent | Number | Percent |  |
| Sex |  |  |  |  |  |  |  |  | <0.001 |
| *Female* | 17,984 | 59.2 | 16,520 | 56.6 | 16,157 | 55.2 | 14,742 | 55.8 |  |
| *Male* | 12,382 | 40.8 | 12,669 | 43.4 | 13,755 | 42.4 | 14,867 | 42.8 |  |
| Race |  |  |  |  |  |  |  |  | <0.001 |
| *American Indian or Alaska Native* | 120 | 0.4 | 144 | 0.5 | 136 | 0.5 | 157 | 0.5 |  |
| *White* | 27,940 | 92.0 | 26,266 | 90.0 | 26,404 | 87.8 | 25,232 | 88.7 |  |
| *Native Hawaiian or Pacific Islander* | 26 | 0.1 | 67 | 0.2 | 101 | 0.2 | 177 | 0.2 |  |
| *Asian* | 371 | 1.2 | 525 | 1.8 | 487 | 1.8 | 509 | 1.8 |  |
| *Black or African American* | 1,909 | 6.3 | 2,187 | 7.5 | 2,784 | 7.3 | 3,534 | 7.4 |  |
| Currently Smoking | 3,794 | 12.5 | 3,641 | 12.5 | 3,998 | 12.2 | 4,362 | 12.3 | <0.001 |
| Dialysis | 77 | 0.3 | 53 | 0.2 | 68 | 0.2 | 65 | 0.2 | 0.311 |
| Platelet <150K | 1,382 | 4.6 | 1,207 | 4.1 | 1,269 | 4.0 | 1,334 | 4.1 | 0.0337 |
| Anemic | 4,252 | 14.0 | 3,969 | 13.6 | 4,101 | 13.3 | 4,517 | 13.4 | <0.001 |
| Age |  |  |  |  |  |  |  |  | <0.001 |
| *0-58* | 6,656 | 21.9 | 7,512 | 25.7 | 8,735 | 25.1 | 10,468 | 25.4 |  |
| *59-65* | 6,751 | 22.2 | 6,702 | 23.0 | 7,276 | 22.4 | 7,269 | 22.6 |  |
| *66-72* | 7,601 | 25.0 | 7,129 | 24.4 | 6,821 | 23.8 | 5,963 | 24.1 |  |
| *73+* | 9,358 | 30.8 | 7,846 | 26.9 | 7,080 | 26.2 | 5,909 | 26.5 |  |
| Cardiovascular Disease | 17,572 | 57.9 | 16,682 | 57.2 | 17,062 | 55.8 | 16,990 | 56.3 | 0.174 |
| Obese | 12,068 | 39.7 | 12,946 | 44.4 | 14,675 | 43.3 | 15,952 | 43.7 | <0.001 |
| Diabetic | 3,594 | 11.8 | 3,477 | 11.9 | 3,558 | 11.6 | 3,756 | 11.7 | 0.0034 |
| Pulmonary Comorbidities | 1,309 | 4.3 | 1,138 | 3.9 | 1,142 | 3.8 | 1,171 | 3.8 | 0.0109 |
| Bleeding Disorder | 665 | 2.2 | 658 | 2.3 | 638 | 2.2 | 694 | 2.2 | 0.34 |
| Steroid or Immunosuppressant use | 1,109 | 3.7 | 1,077 | 3.7 | 1,090 | 3.6 | 1,158 | 3.6 | 0.266 |
| Dyspnea | 1,484 | 4.9 | 1,189 | 4.1 | 1,318 | 4.0 | 1,324 | 4.0 | <0.001 |
| Preoperative Transfusion | 30 | 0.1 | 22 | 0.1 | 39 | 0.1 | 47 | 0.1 | 0.0174 |

**Supplimental Table 1.** Univariate χ ^2^ Analysis of Possible Preoperative Covariates in THA.
