# Supplementary material for: Surgical Duration Implicated in Major Postoperative Complications in Total Hip and Total Knee Arthroplasty: A Retrospective Cohort Study
Source: J Am Acad Orthop Surg Glob Res Rev. 2020 Nov 4;4(11):e20.00043. doi: 10.5435/JAAOSGlobal-D-20-00043 (PMC7643914; doi:10.5435/JAAOSGlobal-D-20-00043)
Supplement: SUPPLEMENTARY MATERIAL [file jagrr-4-e20.00043-s002.docx]

| **Postoperative Complication** | **Quartile 2** | | | | **Quartile 3** | | | **Quartile 4** | | |
| --- | --- | --- | --- | --- | --- | --- | --- | --- | --- | --- |
|  | OR | 95% CI | P-value | | OR | 95% CI | P-value | OR | 95% CI | P-value |
| Septic Shock | 1.13 | 0.49-2.62 | | 0.767 | 2.27 | 1.1-4.7 | 0.0273 | 3.16 | 1.57-6.35 | 0.00127 |
| Surgical Site Infection | 1.32 | 1.01-1.72 | | 0.0453 | 1.66 | 1.29-2.15 | <0.001 | 2.35 | 1.85-3 | <0.001 |
| Deep Wound Infection | 1.1 | 0.73-1.65 | | 0.652 | 1.57 | 1.08-2.28 | 0.0185 | 2.37 | 1.67-3.37 | <0.001 |
| Organ Space Infection | 1.43 | 0.98-2.08 | | 0.0616 | 1.65 | 1.15-2.37 | 0.0062 | 1.89 | 1.33-2.69 | <0.001 |
| Dehiscence | 0.64 | 0.34-1.21 | | 0.169 | 1.37 | 0.82-2.29 | 0.226 | 2.31 | 1.44-3.7 | <0.001 |
| Wound Infection | 1.25 | 1.03-1.5 | | 0.0227 | 1.63 | 1.37-1.95 | <0.001 | 2.27 | 1.92-2.68 | <0.001 |
| Pneumonia | 1.06 | 0.8-1.4 | | 0.707 | 1.05 | 0.8-1.4 | 0.71 | 1.16 | 0.88-1.54 | 0.291 |
| UTI | 1.16 | 0.97-1.4 | | 0.103 | 1.22 | 1.02-1.47 | 0.0296 | 1.49 | 1.24-1.78 | <0.001 |
| Myocardial Infarction | 1.21 | 0.86-1.69 | | 0.268 | 1.08 | 0.76-1.53 | 0.681 | 1.07 | 0.75-1.53 | 0.722 |
| Reintubation | 1.04 | 0.69-1.55 | | 0.855 | 0.96 | 0.64-1.45 | 0.851 | 1.28 | 0.87-1.89 | 0.205 |
| Postop Stroke | 1.83 | 1.05-3.19 | | 0.034 | 1.71 | 0.97-3.01 | 0.0657 | 1.68 | 0.93-3.01 | 0.0839 |
| Postop Transfusion | 1.23 | 1.15-1.33 | | <0.001 | 1.67 | 1.56-1.8 | <0.001 | 3.36 | 3.15-3.59 | <0.001 |
| DVT | 1.11 | 0.85-1.44 | | 0.448 | 1 | 0.76-1.31 | 0.973 | 1.49 | 1.16-1.92 | 0.00189 |
| Cardiac Arrest | 0.59 | 0.3-1.17 | | 0.13 | 1.04 | 0.59-1.86 | 0.883 | 0.89 | 0.48-1.64 | 0.705 |
| VTE | 0.97 | 0.7-1.34 | | 0.847 | 0.97 | 0.7-1.34 | 0.851 | 1 | 0.72-1.38 | 0.995 |

**Supplemental Table 2.** MLR Analysis of the Effect of Surgical Duration on Postoperative Complications in THA.
