# Supplementary material for: Surgical Duration Implicated in Major Postoperative Complications in Total Hip and Total Knee Arthroplasty: A Retrospective Cohort Study
Source: J Am Acad Orthop Surg Glob Res Rev. 2020 Nov 4;4(11):e20.00043. doi: 10.5435/JAAOSGlobal-D-20-00043 (PMC7643914; doi:10.5435/JAAOSGlobal-D-20-00043)
Supplement: SUPPLEMENTARY MATERIAL [file jagrr-4-e20.00043-s003.docx]

| **Preoperative Variable** | **Quartile 1** | | **Quartile 2** | | **Quartile 3** | | **Quartile 4** | | **P-value** |
| --- | --- | --- | --- | --- | --- | --- | --- | --- | --- |
|  | Number | Percent | Number | Percent | Number | Percent | Number | Percent |  |
| Sex |  |  |  |  |  |  |  |  | <0.001 |
| *Female* | 34,203 | 68.3 | 29,103 | 64.0 | 2,397 | 62.3 | 26,082 | 61.9 |  |
| *Male* | 15,872 | 31.7 | 16,390 | 36.0 | 18,321 | 35.1 | 20,929 | 34.9 |  |
| Race |  |  |  |  |  |  |  |  | <0.001 |
| *American Indian or Alaska Native* | 254 | 0.5 | 320 | 0.7 | 286 | 0.7 | 314 | 0.7 |  |
| *White* | 45,585 | 91.0 | 40,394 | 88.8 | 40,736 | 86.5 | 39,056 | 85.9 |  |
| *Native Hawaiian or Pacific Islander* | 127 | 0.3 | 185 | 0.4 | 206 | 0.4 | 327 | 0.4 |  |
| *Asian* | 1,115 | 2.2 | 1,181 | 2.6 | 1,126 | 2.5 | 1,234 | 2.5 |  |
| *Black or African American* | 2,994 | 6.0 | 3,413 | 7.5 | 4,364 | 7.3 | 6,080 | 7.3 |  |
| Currently Smoking | 4,047 | 8.1 | 3,625 | 8.0 | 4,072 | 7.8 | 4,386 | 7.7 | <0.001 |
| Dialysis | 84 | 0.2 | 50 | 0.1 | 79 | 0.1 | 83 | 0.1 | 0.0381 |
| Platelet <150K | 2,193 | 4.4 | 2,110 | 4.6 | 2,205 | 4.5 | 2,372 | 4.5 | <0.001 |
| Anemic | 6,245 | 12.5 | 5,591 | 12.3 | 5,875 | 12.0 | 6,627 | 11.9 | <0.001 |
| Age |  |  |  |  |  |  |  |  | <0.001 |
| *0-58* | 8,417 | 16.8 | 8,127 | 17.9 | 9,403 | 17.4 | 11,555 | 17.3 |  |
| *59-65* | 11,465 | 22.9 | 10,869 | 23.9 | 11,823 | 23.3 | 12,694 | 23.1 |  |
| *66-72* | 14,265 | 28.5 | 12,885 | 28.3 | 12,996 | 27.6 | 12,065 | 27.4 |  |
| *73+* | 15,928 | 31.8 | 13,612 | 29.9 | 12,496 | 29.1 | 10,697 | 29.0 |  |
| Cardiovascular Disease | 33,286 | 66.5 | 30,197 | 66.4 | 31,101 | 64.6 | 31,502 | 64.2 | 0.176 |
| Obese | 30,208 | 60.3 | 28,437 | 62.5 | 30,237 | 60.9 | 31,636 | 60.5 | <0.001 |
| Diabetic | 8,657 | 17.3 | 8,190 | 18.0 | 8,598 | 17.5 | 8,863 | 17.4 | <0.001 |
| Pulmonary Comorbidities | 1,927 | 3.8 | 1,626 | 3.6 | 1,696 | 3.5 | 1,640 | 3.5 | 0.02 |
| Bleeding Disorder | 1,122 | 2.2 | 1,043 | 2.3 | 1,034 | 2.2 | 1,165 | 2.2 | 0.0299 |
| Steroid or Immunosuppressant use | 1,762 | 3.5 | 1,606 | 3.5 | 1,637 | 3.4 | 1,789 | 3.4 | 0.0365 |
| Dyspnea | 3,142 | 6.3 | 2,555 | 5.6 | 2,488 | 5.5 | 2,312 | 5.4 | <0.001 |
| Preoperative Transfusion | 16 | 0.0 | 6 | 0.0 | 10 | 0.0 | 32 | 0.0 | <0.001 |

**Supplemental Table 3.** Univariate χ ^2^ Analysis of Possible Preoperative Covariates in TKA.
