# Supplementary material for: Surgical Duration Implicated in Major Postoperative Complications in Total Hip and Total Knee Arthroplasty: A Retrospective Cohort Study
Source: J Am Acad Orthop Surg Glob Res Rev. 2020 Nov 4;4(11):e20.00043. doi: 10.5435/JAAOSGlobal-D-20-00043 (PMC7643914; doi:10.5435/JAAOSGlobal-D-20-00043)
Supplement: SUPPLEMENTARY MATERIAL [file jagrr-4-e20.00043-s004.docx]

| **Postoperative Complication** | **Quartile 2** | | | **Quartile 3** | | | **Quartile 4** | | |
| --- | --- | --- | --- | --- | --- | --- | --- | --- | --- |
|  | OR | 95% CI | P-value | OR | 95% CI | P-value | OR | 95% CI | P-value |
| Septic Shock | 0.6 | 0.35-1.02 | 0.0602 | 0.4 | 0.22-0.74 | 0.00347 | 0.81 | 0.5-1.33 | 0.41 |
| Surgical Site Infection | 0.88 | 0.7-1.1 | 0.26 | 1.26 | 1.02-1.55 | 0.0288 | 1.71 | 1.41-2.08 | <0.001 |
| Deep Wound Infection | 0.97 | 0.58-1.62 | 0.917 | 2.14 | 1.39-3.28 | <0.001 | 2.58 | 1.7-3.92 | <0.001 |
| Organ Space Infection | 1.14 | 0.78-1.67 | 0.509 | 1.69 | 1.2-2.4 | 0.00285 | 2.26 | 1.62-3.14 | <0.001 |
| Dehiscence | 1.21 | 0.86-1.7 | 0.267 | 1.46 | 1.06-2.01 | 0.0214 | 2.26 | 1.68-3.04 | <0.001 |
| Wound Infection | 0.99 | 0.84-1.17 | 0.943 | 1.44 | 1.24-1.68 | <0.001 | 1.98 | 1.71-2.28 | <0.001 |
| Pneumonia | 0.83 | 0.66-1.03 | 0.0926 | 0.91 | 0.74-1.14 | 0.418 | 0.9 | 0.72-1.12 | 0.327 |
| UTI | 0.91 | 0.79-1.05 | 0.206 | 0.98 | 0.85-1.13 | 0.757 | 0.99 | 0.86-1.15 | 0.921 |
| Myocardial Infarction | 1.01 | 0.74-1.36 | 0.967 | 1.06 | 0.79-1.43 | 0.707 | 1.26 | 0.94-1.68 | 0.124 |
| Reintubation | 0.98 | 0.7-1.36 | 0.9 | 0.84 | 0.59-1.18 | 0.316 | 1.1 | 0.8-1.52 | 0.558 |
| Postop Stroke | 0.77 | 0.5-1.21 | 0.259 | 1.07 | 0.71-1.61 | 0.744 | 0.84 | 0.54-1.32 | 0.453 |
| Postop Transfusion | 0.89 | 0.83-0.95 | <0.001 | 1 | 0.93-1.06 | 0.915 | 1.5 | 1.41-1.6 | <0.001 |
| DVT | 0.93 | 0.8-1.08 | 0.353 | 1.11 | 0.96-1.28 | 0.165 | 1.16 | 1-1.33 | 0.0456 |
| Cardiac Arrest | 0.78 | 0.5-1.24 | 0.295 | 0.76 | 0.48-1.2 | 0.246 | 0.79 | 0.5-1.25 | 0.313 |
| VTE | 1.07 | 0.89-1.28 | 0.462 | 1 | 0.84-1.2 | 0.985 | 1.13 | 0.95-1.35 | 0.175 |

**Supplemental Table 4.** MLR Analysis of the Effect of Surgical Duration on Postoperative Complications in TKA.
